# Post-Training Scopolamine Treatment Induced Maladaptive Behavior in Open Field Habituation Task in Rats

**DOI:** 10.1371/journal.pone.0100348

**Published:** 2014-06-17

**Authors:** Natalija Popović, María Caballero-Bleda, Miroljub Popović

**Affiliations:** 1 Department of Human Anatomy and Psychobiology, Faculty of Medicine, University of Murcia, Murcia, Spain; 2 Research Institute of Aging, University of Murcia, Murcia, Spain; University of L'Aquila, Italy

## Abstract

The effects of scopolamine on memory consolidation are controversial and depend on several factors (i.e. site of administration, time of administration and testing, dose, cognitive task, experimental protocol, specie, strain, etc.). Generally, the range dose of systemic administered scopolamine, used in memory consolidation studies, has varied from 0.05 to 50 mg/kg. However, according to the literature, the most frequently used doses of scopolamine efficient on memory consolidation, are 1 and 30 mg/kg, low and high doses, respectively. In open field habituation studies only lower doses of scopolamine were used to test memory consolidation. Therefore, in the present study we compared the effects of low (1 mg/kg) and high (30 mg/kg) scopolamine dose, on the open field habituation task, in male Wistar rats. Scopolamine was administered immediately after the acquisition task and animals were retested 48 h later on. On the retested day, the ambulation and rearing in the open field decreased in the same manner in all tested groups. In saline- and 1 mg/kg scopolamine-treated animals, the time spent in grooming significantly decreased in the habituation task, while the same parameter significantly increased in animals treated with 30 mg/kg of scopolamine. The defecation rate significantly decreased (control group), maintained (1 mg/kg of scopolamine treated animals) or significantly increased (30 mg/kg of scopolamine treated group) on retention test. In conclusion, the present data suggest that post-training scopolamine administration does not affect locomotion neither exploration in the habituation to a novel environment, but increases defecation and grooming, two behaviours associated with fearful and stressful situations.

## Introduction

Since 1934, when Calvin Hall [1] conceived the open field for the first time, the initial brief exposure of animals to the novel environment has been used to test emotionality in rodents. On the other hand, the re-exposure to the open field has been considered as a habituation to the novel environment, one of the most elementary forms of non-associative hippocampal-dependent learning [2]. Since response to novelty is a complex mechanism that involves several processes including arousal, attention, anxiety, fear and stress-related factors, the habituation should result in the decrease of fear and stress when the animal is re-exposed to the test situation. Although habituation is commonly measured by evaluating the decrease of exploratory behaviour when the environment start to be familiar [3], other parameters can be also used (e.g., grooming, defecation, sitting) [4]. Contrary to habituation, sensitization is a non-associative learning in which the re-exposure to the initial stimulus increases the initial behavioural response.

Systemic post-training scopolamine treatment, a nonselective muscarinic receptor antagonist, disrupted mice habituation (at 10 mg/kg but not at 1 and 3.2 mg/kg) in the nose-poke test [5], open field task (at 2 mg/kg but not at 0.1 mg/kg) [6] and activity cage test (at 0.2–0.8 mg/kg) [7]. In rats, the habituation to odor is impaired by post-training scopolamine treatment (at 0.5 and 1 mg/kg) [8]. On the other hand, post-training scopolamine treatment at 0.75 mg/kg, preserved rat's memory consolidation in the open field habituation task [9]. In above mentioned studies only locomotion and/or exploration were evaluated as habituation parameters. Having in mind that two behaviours associated with fearful and stressful situations, grooming and defecation, have not being considered in the above mentioned reports, the first aim of the present study was to evaluate the effects of scopolaminés post-training treatment on both emotional and locomotors/exploratory components of the open field habituation task.

The effect of high doses of scopolamine on memory consolidation has been tested in fear conditioning [10] and passive avoidance [11] tasks. The results from these studies indicated that scopolamine post-training treatment in a dose of 50 mg/kg changed neither tone nor context fear conditioning in rats, while the dose of 30 mg/kg impaired memory consolidation of the passive avoidance task in mice. The effect of high doses of scopolamine on memory consolidation has not been tested in the open field habituation task. Since the data from the literature indicate that the most frequently used doses of scopolamine efficient on memory consolidation studies are 1 and 30 mg/kg, low and high dose respectively, given immediately after the acquisition task [12], the second aim of the present study was to compare the effect of these doses on the open field habituation.

## Material and Methods

### Experimental Animals

Experiments were carried out on male Wistar rats, weighing 200–250 g. The animals were housed in standard Macrolon cages on sawdust bedding. They were kept in an air-conditioned room (20±1°C), at 30% humidity and under a 12 h light/12 h dark cycle (lights on from 08∶00–20∶00 h). Food and tap water were available *ad libitum*. One week before the experimental procedure, the rats were handled daily for five minutes each. The behavioural tests were performed during the light period (16∶00–20∶00 h).

All procedures related to the animal maintenance and experimentation were in accordance with the European Communities Council Directive of November 24, 1986 (86/609/EEC) and the guidelines issued by the Spanish Ministry of Agriculture, Fishing and Feeding (Royal Decree 1201/2005 of October 21, 2005) and were approved by the Animal Ethics Committee of University of Murcia. Efforts were made to minimize the number of animals used, as well as their suffering.

### Drugs

The saline solution of scopolamine hydrobromide (Sigma, St. Louis, MO) was administered intraperitonelly at the dose of 1 mg/kg or 30 mg/kg. Control animals were treated with physiological saline in dose of 1 ml/kg body weight.

### Open field test

The open field test was performed in a square white plywood box (100×100×40 cm). The floor was divided into 25 (20×20 cm) squares. On day 1, the rats were initially placed at one of the four corners of the box and their behaviour was monitored during 10 min. After that, the rats were removed from the open field, drug administered and returned to their home cage. Forty eight hours later, the retention test was given. In the open field test, the ***ambulation in the board area*** (number of outer squares entered), the ***ambulation in the central area*** (number of inner squares entered), the ***number of rearing*** (standing on hind legs, with or without contact with the sides of the arena), the ***time spent frozen*** (time that animal spent immobile), the ***time spent in grooming*** (time that animal spend licking, scratching or cleaning any part of its head or body) and the ***defecation*** (number of fecal boli deposited) were recorded. The open field test was performed under 300 lux light intensity and recorded using a video camera to enable subsequent evaluation. The apparatus was cleaned with 70% ethanol before each animal was tested. The eight animals were assigned in each tested group.

### Statistical analysis

The statistical analysis was made using the SPSS 19.0 statistical package. The data are presented as mean ± standard error of the mean (S.E.M). The data were analyzed with the General Linear Model (GLM) repeated measures analysis. If the GLM showed significant differences between groups, a post hoc analysis was performed. The group differences on acquisition trial were analyzed by two-tailed Student's t-test for independent samples. The two-tailed Student's t-test for paired-samples was used for comparison of the data between the acquisition and the retention trial. Differences were considered statistically significant if p<0.05.

## Results

**Figure 1:**
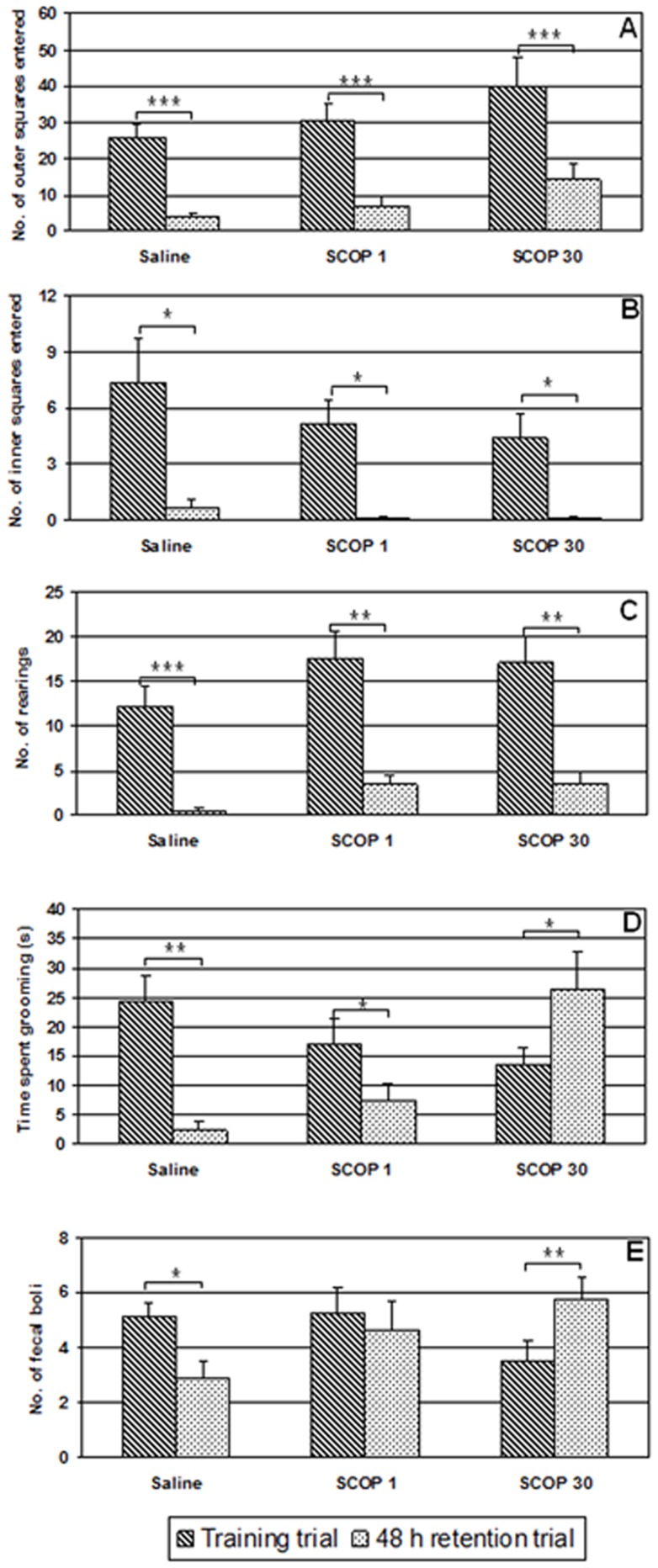
Effect of 1/kg and 30 mg/kg of scopolamine (SCOP-1; SCOP-30, respectively), administered i.p. immediately after the acquisition trial, on ambulation in the board area (A), ambulation in the central area (B), number of rearing (C), time spent in grooming (D) and defecation (E), in the open field habituation task. The data are presented as mean ± standard error of the mean (S.E.M). *p<0.05; **p<0.01; ***p<0.001 vs. acquisition trial.

Only one animal from the saline treated group and two animals from the scopolamine treated groups displayed freezing behaviour (data not showed). The rest of the data from the open field test are presented in Figure 1.

The GLM repeated measure analysis showed a significant *effect of time* on ambulation in the board area (F_(1)_ = 71.139, p<0.001), ambulation in the central area (F_(1)_ = 36.781, p<0.001), number of rearing (F_(1)_ = 69.097, p<0.001), time spent grooming (F_(1)_ = 5.826, p<0.05) but not on defecation (F_(1)_ = 0.300, p>0.05). There was no significant *effect of group* on ambulation in the board area (F_(2)_ = 2.905, p>0.05), ambulation in the central area (F_(2)_ = 0.916, p>0.05), number of rearing (F_(2)_ = 2.045, p>0.05), time spent grooming (F_(2)_ = 1.374, p>0.05) and defecation (F_(2)_ = 0.430, p>0.05). There was significant *effect of interaction time x group* on the time spent grooming (F_(2)_ = 15.719, p<0.001) and on defecation (F_(2)_ = 11.971, p<0.001), but not on ambulation in the board area (F_(2)_ = 0.157, p>0.05), ambulation in the central area (F_(2)_ = 0.683, p>0.05) and number of rearing (F_(1)_ = 0.223, p>0.05).

In the acquisition trial of the open field test there were no significant differences between groups in the ambulation in the board area, ambulation in the central area, number of rearing, time spent in grooming and defecation rate. The two-tailed Student's t-test for paired-samples showed that ambulation in the board (t = 6.677, d.f. = 7, p<0.001; t = 9.881, d.f. = 7, p<0.001; t = 3.474, d.f. = 7, p<0.01, respectively) (Fig. 1A), ambulation in the central area (t = 3.441, d.f. = 7, p<0.01; t = 4.023, d.f. = 7, p<0.01; t = 3.362, d.f. = 7, p<0.05, respectively) (Fig. 1B) and the number of rearing (t = 5.276, d.f. = 7, p<0.001; t = 4.714, d.f. = 7, p<0.01; t = 4.637, d.f. = 7, p<0.01, respectively) (Fig. 1C) significantly decreased on retention day in both the saline- and scopolamine-treated (1 and 30 mg/kg) animals. On the retention day, the time spent grooming significantly decreased in the groups treated with saline (t = 4.376, d.f. = 7, p<0.01) and scopolamine in the dose of 1 mg/kg (t = 3.104, d.f. = 7, p<0.05) while animals treated with scopolamine in the dose of 30 mg/kg showed increase in grooming behaviour (t = −2.615, d.f. = 7, p<0.05) (Fig. 1D). The defecation rate significantly decreased in the saline-treated animals but not in the scopolamine-treated (1 mg/kg) group (t = 3.000, d.f. = 7, p<0.05; t = 1.049, d.f. = 7, p>0.05, respectively) (Fig. 1E). Similarly to the grooming behaviour, the animals treated with scopolamine in the dose of 30 mg/kg showed increase in the defecation rate on the retention day (t = −3.631, d.f. = 7, p<0.01) (Fig. 1E).

## Discussion

Evidence exists that either during initial exposure and re-exposure to the open field, the extracellular level of hippocampal acetylcholine (ACh) is elevated in rats [13], [14]. The increase of extracellular levels of hippocampal ACh correlates with the level of rearing on the initial exposure to the novelty, but not on the retention trial [13]. On the other hand, hippocampal ACh is positively related to the locomotor activity during the retention trial, but not during the initial trial [14] or neither locomotor activity nor grooming behaviour correlate with hippocampal ACh levels [13]. On the light of those data, it is not surprising that scopolamine pre-training administration impairs habituation of rearing, but not ambulation, in the open field test [15]–[17]. On the other hand, scopolamine pre-retrieval exposure impairs habituation in ambulation but not in rearing [16]. In addition, scopolamine pre-training administration increases the fear response on the retrieval session, as it is evidenced by increase of defecation [17].

The effect of scopolamine on memory consolidation of habituation in the open field has not been extensively studied. Taking into account that high levels of acetylcholine in the hippocampus are necessary for the acquisition of new information, while low levels are required for memory consolidation [18]–[21], it could be expected that the post-training scopolamine treatment may facilitate the open field habituation. The present study showed that scopolamine in the dose of 1 and 30 mg/kg did not interfere with the habituation of both ambulation and rearing in rats. Our results are in agreement with previous studies reporting that post-training scopolamine treatment in rats decreased ambulation and rearing on 24 h open field habituation trial [9]. However, the decrease of rearing was more pronounced in control animals than in those treated with scopolamine [9]. In contrast to our results, post-training systemic scopolamine treatment at the dose of 2 mg/kg, but not 0.1 mg/kg, disrupted the habituation of ambulation in the open field task, in mice [6]. Further, the data from another hippocampal dependent tasks indicated that low (1 mg/kg) and high (50 mg/kg) doses of scopolamine did not affect memory consolidation of context fear conditioning in rats [11], while only high dose (30 mg/kg) impaired passive avoidance response in mice [12]. These discrepancies may be due to different factors, such as: specie differences (mice vs. rat), learning task, shape of the open field box (rectangular vs. squared), illumination intensity (with vs. without light focus), duration of the experiment, doses used and time interval between acquisition and retention trial.

In contrast to locomotion and exploration, the scopolamine dose-related lack of grooming and defecation habituation was found in our study. Namely, on the retention trial, in saline and scopolamine (1 mg/kg)-treated animals the grooming behaviour was significantly decreased while the same behaviour significantly increased at higher dose scopolamine-treated animals. In relation to defecation, only animals treated with saline displayed significant habituation (decrease of defecation rate). Conversely, scopolamine-treated animals maintained (1 mg/kg) or increased (30 mg/kg) the defecation level, suggesting the fear perseverance in those animals.

In the previously cited studies as well as in the present study, the systemic administration of scopolamine was performed immediately after the learning trial. When scopolamine was injected into the core of the nucleus accumbens, immediately after the first exposure to the open field, it impaired habituation of rearing and locomotion at the two lower doses, 0.1 and 1.0 µg [22]. However, at the higher dose of 10.0 µg only locomotion was impaired. In contrast to that, when the higher dose was injected with a delay of 5 h after the learning trial, habituation of rearing was impaired while habituation of locomotion was preserved. Further studies are needed to evaluate whether delayed systemic post-training scopolamine treatment will differently affect, in dose dependent manner, emotional and locomotors/exploratory components of the open field habituation task.

It has been found that if two novel learning tasks are sequentially acquired, scopolamine selectively impairs the acquisition of the second learning situation, though without affecting either memory consolidation or reconsolidation of the first one [23]. Therefore, it could be hypothesized that the lack of grooming and defecation habituation, two behaviours associated with fearful and stressful situations [24]–[26], could be due to scopolaminés impairment of attention to new information, then revealing a stressful situation (exposition to a novel environment) on the training day, which leads the animal to the maladaptive behaviour of the retention day.

## Conclusion

As far as we know, the present data represent the first demonstration that post-training scopolamine administration produces a dual effect on habituation in the open field task: even though memory consolidation of locomotion neither exploration are affected, the emotionality response (defecation and grooming) is increased, giving a new insight of the importance of the cholinergic system in the behavioural sensitization to novelty stress.
